# Brief Overview of Approaches and Challenges in New Antibiotic Development: A Focus On Drug Repurposing

**DOI:** 10.3389/fcimb.2021.684515

**Published:** 2021-05-17

**Authors:** Natalie K. Boyd, Chengwen Teng, Christopher R. Frei

**Affiliations:** ^1^ College of Pharmacy, The University of Texas at Austin, San Antonio, TX, United States; ^2^ Long School of Medicine, University of Texas Health San Antonio, San Antonio, TX, United States; ^3^ Department of Clinical Pharmacy and Outcomes Sciences, College of Pharmacy, The University of South Carolina, Columbia, SC, United States; ^4^ Research Department, South Texas Veterans Health Care System, San Antonio, TX, United States; ^5^ Pharmacy Department, University Health System, San Antonio, TX, United States

**Keywords:** drug repurposing, antibiotics, drug development, translational science, reverse translation

## Abstract

Drug repurposing, or identifying new uses for existing drugs, has emerged as an alternative to traditional drug discovery processes involving *de novo* synthesis. Drugs that are currently approved or under development for non-antibiotic indications may possess antibiotic properties, and therefore may have repurposing potential, either alone or in combination with an antibiotic. They might also serve as “antibiotic adjuvants” to enhance the activity of certain antibiotics.

## Drug Discovery: Overview of Process

Traditional drug discovery strategies aim to identify the next new chemical or molecular entity that possesses a novel mechanism of action. For any promising new compound, the path from initial discovery to market launch is sluggish, costly, and fraught with a multitude of barriers. Moving a new drug from pre-clinical phases to market generally requires a minimum timeframe of 10-12 years and over $2 billion in resources (DiMasi, 2014; DiMasi et al., 2016). Additionally, the probability of success is low, with only 1-2 drugs from an initial 10,000 compounds reaching Federal Drug Administration (FDA) approval. Following target identification and validation, high-throughput screening (HTS) assays are developed and run against compound libraries to generate ‘hits’, which are the compounds that demonstrate the desired activity or interaction with the target of interest. Each hit series undergoes additional screening and/or chemical modifications to become more ‘druggable’ lead compounds before *in vitro* and *in vivo* pharmacokinetic testing is performed in animal models (preclinical). Generally, only a handful of drug candidates from the initial 10,000 compounds enter clinical trials. Success rates for drugs entering Phase I clinical trials have approximately 10% chance of gaining FDA approval for the desired indication (Mullard, 2016).

## Antibiotic Drug Development: A Historical Perspective

Milestones of antibiotic discovery and development can offer insights into future solutions. The pre-antibiotic era bears striking resemblance to circumstances of today, regarding a need for: 1) novel, effective antibiotics, 2) large scale collaboration, and 3) efficient processes/timelines for antibiotic approvals.

### Penicillin: A Landmark “Bench to Bedside” Breakthrough

The discovery of penicillin in 1928 is regarded as one of the most significant medical and scientific breakthroughs in history (Ligon, 2004a; Ligon, 2004b; Kardos and Demain, 2011; Lobanovska and Pilla, 2017). It represents one of history’s earliest examples of translating a scientific discovery into medicine. The story of how penicillin was developed is as important as the discovery of the drug itself. Overcoming the major barriers during that time helped establish methods that led to next-generation penicillins and development of other antibiotic classes (Kardos and Demain, 2011; Lobanovska and Pilla, 2017).

When a fungal contaminant (*Penicillium notatum*) on a petri dish was found to produce a potent substance that inhibited growth of *Staphylococcus aureus*, Alexander Fleming had unknowingly discovered a life-saving antibiotic. He published his findings the following year (Fleming, 1929), but surprisingly, the world did not take notice right away. Furthermore, there were problems with the drug itself. Penicillin was chemically unstable and difficult to isolate from the mold, raising serious doubts about its potential as a therapeutic agent.

More than ten years later, in 1939, a group of scientists at Oxford University (Howard Florey, Ernest Chain, Norman Heatley, and Edward Abraham) took on this challenge, and successfully developed a procedure for isolating and purifying penicillin (Chain et al., 2005), thereby enabling extraction of sufficient material to conduct *in vivo* efficacy studies (Chain et al., 1993). Clinical trials began in 1941, demonstrating drug stability and efficacy against *Streptococcus pyogenes* and *Staphylococcus aureus*, with no signs of toxicity (Ligon, 2004a; Ligon, 2004b; Lobanovska and Pilla, 2017).

With World War II underway, there was an established need for penicillin use in wounded soldiers and civilians (Fraser, 1984; Ligon, 2004a; Ligon, 2004b); thus, creating an intense sense of urgency for large-scale production. Funding, however, was limited in England for a task of this size, leading the Oxford team to relocate to the United States for assistance. Collaborative efforts soon began at an unprecedented level between the United States and United Kingdom that included academic and government entities teaming up with multiple pharmaceutical companies (including Merck, Squibb, Lilly, and Pfizer to start). Through their combined resources and expertise, they developed new procedures for the purification and mass production of penicillin - and just in time for D-Day (1944) (Swann, 1983; Fraser, 1984). These were ground-breaking techniques (i.e. submerged fermentation, microbial strain production, mutational strain improvement) representing a combination of scientific disciplines including microbiology, biochemistry, and chemical engineering, that quickly became integral for the development of subsequent antibiotics (Richards, 1964).

Shortly after the war in 1946, penicillin became widely available by prescription, which revolutionized medicine. Providers were able to, for the first time; effectively treat previously incurable diseases such as rheumatic fever, scarlet fever, syphilis, severe wounds, and infections with aggressive pathogens such as *Staphylococcus* or *Streptococcus* spp (Dowling and Lepper, 1951; Armstrong et al., 1999; Kardos and Demain, 2011; Aminov, 2017). Alexander Fleming’s serendipitous discovery of penicillin was the breakthrough of the century; however, it took an international collaboration composed of government, academia, and industry scientists to translate this discovery into one of the most important medical treatments in history.

### The Antibiotic Era

The drug discovery landscape was forever changed after the arrival of penicillin. Not only did it save thousands of lives, it also ushered in an era of natural products discovery (Wright, 2014; Moloney, 2016). Building on the work of Fleming, microbiologist Selman Waksman sought to find more sources of antibiotic-producing microbes from soil. His approach involved the screening of soil-derived bacteria (mostly *Actinomycetes* spp.) against susceptible test organisms and evaluating zones of inhibited growth on an overlay plate (Schatz et al., 2005). This method is similar to Fleming’s discovery of penicillin; however, Waksman applied a more systematic, deliberate screening approach, while Fleming’s discovery of an antibiotic-producing mold was accidental. This new screening approach, otherwise known as the ‘Waksman platform’ led to the discovery of an important antibiotic streptomycin, which exhibited *in vitro* activity against Gram-positive and Gram-negative bacteria (Jones et al., 1944). Though penicillin was highly effective and in frequent use at the time, its antibacterial activity was primarily limited to Gram-positive bacteria. Streptomycin, the first of the aminoglycoside antibiotic class, was also the first drug with activity against *Mycobacterium tuberculosis*.

After the successful launch of streptomycin, the Waksman platform quickly became the quintessential tool for antibiotic discovery at the time, and ultimately the most successful and widely adopted antibiotic discovery platform to date. Discovery of other antibiotics occurred shortly thereafter, and continued over the next 20 years, famously referred to as the ‘golden age’ of antibiotics (Lewis, 2013; Lyddiard et al., 2016). In fact, the bulk of antibiotics in use today are from natural products or their semisynthetic derivatives that were discovered by this method of mining through soil-derived compounds (Moloney, 2016; Mohr, 2016; Katz and Baltz, 2016). Vancomycin, clindamycin, rifampin, tetracycline, and daptomycin are among a few important natural product antibiotics discovered during this era that remain in use today (**Table 1**).

**Table 1:** Antibiotics derived from natural products (Lewis, 2013; Wright, 2014).

Antibiotic class	Example of clinically used drugs	Biological target
β-lactam	Penicillins: amoxicillin, ampicillin, piperacillin, cephalosporins: cephalexin, cefaclor, ceftazidime, Carbapenems: imipenem, meropenem	Peptidoglycan synthesis; transpeptidases
Glycopeptide	Vancomycin	Peptidoglycan synthesis; binding to acyl-D-Ala-D-Ala
Macrolide	Erythromycin, clarithromycin, azithromycin	Ribosome; blocks peptide exit tunnel in the large subunit
Lincosamide	Clindamycin	Ribosome; blocks peptide exit tunnel in the large subunit
Aminoglycoside	Gentamicin, tobramycin, amikacin	Ribosome; impairs cognate aminoacyl-tRNA recognition
Streptogramin	Synercid (quinupristin + dalfopristin)	Ribosome: inhibits peptidyl transfer, blocks peptide exit tunnel in large subunit
Tetracycline	Doxycycline, minocycline	Ribosome: inhibits aminoacyl-tRNA transfer, blocks peptide exit tunnel in small subunit
Rifamycin	Rifampin	RNA polymerase
Lipopeptide	Daptomycin	Cell membrane
Cationic peptide	Colistin	Cell membrane

### Antibiotic Innovation Gap

During the golden age of antibiotics, from 1940 through 1960s, the antibiotic development pipeline flourished (Walsh and Wencewicz, 2014). In fact, the rapidity of new antibiotics discovered at the time appeared to be outpacing the spread of antibiotic resistance. However, the majority of antibiotics developed during this period were through natural product discovery, a few synthetic antibiotic classes or “scaffolds” were also developed with success and remain in used today: fluoroquinolones (ciprofloxacin, levofloxacin), sulfonamides (sulfamethoxazole), oxazolidinones (linezolid), and nitroimidazole (metronidazole) (Wright, 2014; Lewis, 2017). Rapid advances in biotechnology gave rise to HTS in the early 1990s (Kubinyi, 1995). Plus, with the advancements in medicinal chemistry, molecular biology, and arrival of genomic tools, the pharmaceutical industry was seemingly more equipped than ever to discover the next wave of novel antibiotic compounds. Expectations were high for productivity as the industry moved away from laboriously mining soil for naturally-occurring compounds, opting instead for target-based HTS of synthetic compounds (Silver, 2011; Lewis, 2013). However, these high-tech platforms yielded only disappointing returns. What occurred instead was nearly a 40-year innovation gap. After the introduction of nalidixic acid in 1962, no new structural classes of antibiotics were developed again until linezolid in 2000 (Walsh, 2003; Fischbach and Walsh, 2009).

A combination of several important factors is likely to blame for the antibiotic innovation gap or discover void lasting several decades:

#### Collapse of the Waksman Platform

This drug discovery platform was a success for approximately 20 years. Unfortunately, mining through soil microbes eventually led to frequent re-isolation or rediscovery of known compounds (Katz and Baltz, 2016). After yielding diminished returns, the platform was abandoned. Still, many experts now advocate for a revival of this platform, as synthetic approaches have been unable to replace the success of natural product drug discovery. Furthermore, soil and marine environments may still be promising untapped sources for antibiotic compounds. Metagenomic analyses have shown that 99% of bacteria from soil and marine samples are “uncultured,” meaning they do not grow under normal laboratory conditions (Rappe and Giovannoni, 2003; Schloss and Handelsman, 2004). Recently, investigators unveiled the discovery of a new antibiotic, teixobactin (Ling et al., 2015; Fiers et al., 2017), using a method similar to the Waksman platform, but with a modified technique for isolating and growing uncultured bacteria (Nichols et al., 2010).

#### Golden Age of Medicinal Chemistry

Much of the focus in the pharmaceutical industry during the 1960s and 1970s shifted from novel discovery of compounds to the chemical tailoring of existing antibiotics to create successive generations of antibiotics (2nd, 3rd, 4th, etc.). This has been important for improving the efficacy and/or pharmacological properties of antibiotics but has not lead to any new molecular entities or novel antibiotic scaffolds (Walsh, 2003; Aminov, 2017; Lewis, 2017).

#### Adherence to Lipinski ‘Rule of Five’

Poor absorption was a major source of attrition during drug discovery in the early 1990s (Kola and Landis, 2004). Renowned medicinal chemist, Christopher Lipinski, along with his team, sought to determine the physiochemical properties of compounds that best predict absorption and advancement to clinical stages of development. Lipinski analyzed properties of compounds that had emerged from Phase I and entered Phase II clinical studies, accessed through the World Drug Index (WDI) and United States Adopted Name (USAN) databases (Lipinski et al., 2001). More than 90% of compounds that reached Phase II status had the following physiochemical parameters: i) molecular weight < 500, ii) number of hydrogen-bond donors < 5, iii) number of hydrogen-bond acceptors < 10, and iv) calculated octanol-water partition coefficient < 5. These characteristics, which became known as Lipinski’s rule of five are associated with solubility and permeability, and therefore increased absorption (Lipinski, 2000). This rule-based approach for synthesizing or screening new compounds was widely adopted by the pharmaceutical industry. Antibiotics, however, are unique molecules, and have always been an exception to the Lipinski rules. Unlike drugs developed for other therapeutic areas, antibiotic drug candidates must be able to penetrate bacterial cells, and not just human cells. Lipinski’s rules do not account for this critical physiochemical property (O’Shea and Moser, 2008; Lewis, 2013). In fact, the widespread use of Lipinski’s ‘rule of five’ may have inadvertently selected against discovery of new antibiotic compounds.

#### Phenotypic Versus Target-Based Screens

Antibiotics during the Golden Age were discovered empirically using *in vitro* growth inhibition assays, in which phenotypic endpoints were recorded as bacterial ‘growth’ or ‘no growth’ (Waksman et al., 1946; Ligon, 2004a; Ligon, 2004b; Moloney, 2016; Katz and Baltz, 2016). Mechanisms of action were usually determined later, often many years after approval - a significant downside to using traditional whole-cell phenotypic assays. Following the arrival of genomics, bioinformatics, and high throughput screening, drug screening strategies shifted from phenotypic to molecular target-based platforms, thereby enabling target identification and validation of important disease-related targets (Flordellis et al., 2006; Lewis, 2013). A target-based method involves the *in vitro* interaction between a drug candidate and a defined/validated target (e.g. enzyme or receptor) in a cell-free system. Other distinguishing characteristics between phenotypic and target-based screening is described in **Table 2**.

**Table 2:** Comparing target-based and phenotypic-based screens (Swinney and Anthony, 2011; Zheng et al., 2013; Bell et al., 2015; Wagner, 2016; Moffat et al., 2017).

	Target-based	Phenotypic-based
Assay format/characteristics	Often recombinant proteins (cell-free system) or cells over-expressing the target of interest High throughput Screen measures drug effect on target of interest Hypothesis-driven (target known at starting point)	Whole cells (e.g. cell lines) or organisms (e.g. bacteria) are used Low to medium throughput Screen measures biological effect on cells, tissue, or organism Does not rely on hypothesis
Advantages	Target or mechanism identified	Screen hits are biologically active Screen activity may translate better to human disease
Disadvantages	Screen hits may not be biologically active	Additional study needed to determine target or mechanism of action

Perceived at the time as the more sophisticated and more promising screening platform for anti-infective research and development, target-based screens soon became widely favored in industry. However, this highly anticipated method was met with disappointing results - no new antibiotics emerged from these platforms. Payne et al. published a highly influential article, in which the authors provided candid insight on experiences with target-based screening platforms at GlaxoSmithKline (GSK) (Payne et al., 2007). Between 1995 and 2001, a total of 67 HTS campaigns using genomic-derived antibiotic targets were run against a large compound collection, consisting of 260,000 to 530,000 compounds. This was an unprecedented amount of effort and resources for screening only one single therapeutic area. Only 16 ‘hits’ were identified, five of which became ‘lead’ compounds; but ultimately none of them reached clinical trial phases. Payne et al. concluded that whole-cell phenotypic assays, rather than target-based, are more likely to produce successful leads (Payne et al., 2007). The authors discussed several possibilities for the poor performing results, one of which is an inability to translate *in vitro* activity observed from target-based assays to activity that occurs with live bacterial cells. Target-based screening can produce many ‘hits.’ However, if these compounds cannot overcome the permeability barriers and tendencies for efflux pump activity in bacteria, then none of them, not one single hit, will progress to a lead compound (Livermore and British Society for Antimicrobial Chemotherapy Working Party on The Urgent Need: Regenerating Antibacterial Drug D, Development, 2011; Aminov, 2017; Lewis, 2017; Moffat et al., 2017; Singh et al., 2017). According to Dr. Kim Lewis, Ph.D., Distinguished Professor of Biology and Director of Antimicrobial Discovery Center at Northeastern University, “…simply doing more high-throughput screening or adding yet another target to the long list of potential ones will not do” (Lewis, 2017).

Although the cell permeability hurdle was specific to bacterial cells, the lower productivity from target-based screens does not appear to be limited to the development of antibiotics. An analysis of FDA drug approvals between 1999 and 2008 revealed a higher number of first-in-class compounds (i.e. new molecular entities) discovered through phenotypic screening compared to molecular target-based approach (Swinney and Anthony, 2011). From a total of 50 new in-class drugs, 28 (56%) were discovered using a phenotypic approach, while 17 (34%) were from target-based methods. One area in which target screening appears to be more successful, however, is in the field of cancer. Between 1999 and 2013, 31 of the 48 first in-class oncology drugs were discovered through target-based screens, 21 of which were kinase inhibitors (Moffat et al., 2014).

Despite having fallen out of favor more than two decades ago, and replaced with molecular target-based platforms, phenotypic screening has been undergoing a resurgence (Zheng et al., 2013; Wagner, 2016; Moffat et al., 2017). The ideal screening strategy to improve productivity in antibiotic drug discovery, however, is one that combines advantages of both phenotypic and target screening, while circumventing their limitations (Zheng et al., 2013; Farha and Brown, 2015; Matano et al., 2016). This can be accomplished through a number of ways. One approach is through the use of a parent wildtype bacterial strain paired with a mutant or modified strain, in which a specific target or mechanism of interest has been altered (Farha and Brown, 2015). Comparing the *in vitro* response of a wildtype/mutant pair to drug candidates can preferentially reveal compounds that inhibit a target or pathway. This method allows for a more hypothesis-driven phenotypic approach, for which the screen hits are biologically active, and the mechanism of action can also be deduced. There are increasing reports of using this integrated strategy, some referring to it as target-, pathway-, or mechanism-based whole cell screens (Testa and Johnson, 2012; Gengenbacher and Dick, 2015; Matano et al., 2016; Bonnett et al., 2016).

#### High-Risk Investments

Antimicrobial research and development (R&D) programs were becoming unattractive investments. After the ‘high-tech’ target-based platforms failed to produce new antibiotics, the anti-infective divisions within the pharmaceutical industry began shutting down (Payne et al., 2007; Tommasi et al., 2015; Lewis, 2017). Turning a profit may take years, possibly not until final years of the patent life, depending on the circumstances (Fisher and Mobashery, 2016). Furthermore, antibiotics in general tend to have short durations of therapy (≤ 14 days). In contrast, chronic conditions, such as diabetes or hypertension may require daily treatment for many years, if not for life.

Pharmaceutical companies estimate risk/benefit and profitability of a developing product using a metric known as net present value (NPV). This is a summation of Research and Development (R&D) costs and expected value of future revenue (Sciarretta et al., 2016). A threshold target NPV of $200 million is recommended for an antibiotic to be an attractive investment and comparable to other therapeutic classes (Sharma and Towse, 2011). However, the predicted NPV of an antibiotic is estimated at negative $50 million, meaning the developmental costs would exceed projected earnings (Sharma and Towse, 2011). Among the more recently launched antibiotics, Avycaz (ceftazidime/avibactam) and Teflaro (ceftaroline fosamil) had the highest sales at ~ $80 and ~$50 million, respectively, two years post-launch. Meanwhile, other popular non-antibiotic drugs had sales ranging from $500 million to over $1 billion (Fernandes and Martens, 2017). Thus, even for antibiotics that bring in revenue, the return of investment is low compared to other popular ‘blockbuster’ treatments for other conditions.

### Emergence of Resistance

Antibiotic resistance is an extremely complicated problem. The urgency to develop new antibiotics is almost entirely driven by escalating resistance rates (Theuretzbacher, 2011). The first sign of penicillin resistance was observed in 1940, several years before penicillin was available for widespread use (1945), when a penicillin-inactivating enzyme (penicillinase) was discovered in an *E. coli* strain (Abraham and Chain, 1988). In 1942, penicillin resistance was noted in four clinical strains of *S. aureus*, also by a penicillinase (Rammelkamp and Stolzer, 1961). Unfortunately, this was only the beginning. As each new antibiotic was launched into market, reports of resistance followed shortly thereafter. Over time, this pattern began occurring in a variety of bacterial pathogens, spanning several decades. Today, there is no shortage of antibiotic resistant bacteria, but there is a shortage of effective treatment options.

Until better control measures are in place and more novel antibiotics are available, the threat of resistance will loom, putting an expiration date on each and every antibiotic in use.

### Addressing the Unmet Clinical Need

The emergence and spread of resistant bacteria, coupled with the paucity of new antibiotics, has evolved into a global health crisis (French, 2010; Lushniak, 2014; Rossolini et al., 2014; Brown and Wright, 2016; Martens and Demain, 2017). The Centers for Disease Control and Prevention (CDC) estimated that two million patients per year in the U.S. have infections associated with drug-resistant bacteria and 23,000 die annually as a result (Centers for Disease Control and Prevention, 2013). If antimicrobial resistance continues its current trajectory, an estimated 10 million deaths worldwide are predicted by 2050 (surpassing cancer) (Review on Antimicrobial Resistance, 2015). Industry sponsors, regulatory agencies, and organizations at the national and international level are taking action to overcome hurdles that led to a dry antibiotic pipeline. Just as the Oxford group discovered during their wartime efforts of mass-producing penicillin, public-private collaborations are critical to successfully revive the antibiotic pipeline and bring new antibiotics to patients in need (Luepke and Mohr, 2017; Luepke et al., 2017).

In an effort to improve regulatory processes for drug approvals, the FDA developed four expedited drug review pathways (**Table 3**) for the treatment of life-threatening/serious or rare conditions: i) accelerated approval, ii) priority review, iii) fast track, and iv) breakthrough therapy (Guidance for industry: expedited programs for serious conditions – drugs and biologics, 2014; Hwang et al., 2017). Bacterial infections were not specifically addressed in these pathways. That changed, however, in 2012 when the Generating Antibiotic Incentives Now (GAIN) act was signed into law. Under the GAIN act, industry sponsors can petition the FDA for a Qualified Infectious Disease Product (QIDP) designation, defined as an antibacterial or antifungal drug for human use intended to treat serious or life-threatening infections. Antibiotics with QIDP designation can receive both fast track and priority review status (Brown, 2013). The FDA is expected to have frequent meetings and written communications with the sponsor and provide guidance along the way on developing pathogen-focused antibiotics. QIDP antibiotics are also eligible for five additional years of exclusivity, which allows the sponsor a longer timeframe to recoup development costs.

**Table 3:** FDA expedited regulatory pathways (Darrow et al., 2014; Kesselheim and Darrow, 2015; Sinha and Kesselheim, 2016).

Program	Year initiated	Criteria or Data required	Key characteristics
Accelerated approval	1992	− Treatment of serious condition − Early evidence showing advantage over existing therapies	− FDA approval based on surrogate endpoint − Approval granted on conditional basis, post-approval trial required to confirm clinical benefit
Priority review	1992	− Treatment of serious condition or drug is designated as QIDP − Improvement in safety or effectiveness over existing therapies	− Shorter FDA review timeline (six vs. ten months)
Fast track	1997; 2012*	− Treatment of serious condition or drug is designated as QIDP − Preclinical or clinical evidence demonstrating potential to address unmet medical need.	− Rolling NDA review − More frequent written communication from FDA
Breakthrough therapy	2012	− Treatment of serious condition − Demonstrates substantial improvement over existing therapies on one or more clinically important endpoints	− Intensive FDA guidance throughout development to generate additional safety and efficacy data − Largely oncology and orphan diseases

*Fast track designation was amended by the FDA Safety and Innovation Act (FDASIA) in 2012 to include the GAIN Act.

NDA, new drug application; QIDP, Qualified Infectious Disease Product; FDA, Food and Drug Administration.

In addition to expedited approval programs, another pathway, the Limited Population Antimicrobial Drug (LPAD) pathway, was signed into law in 2016 as a provision to the 21st Century Cures Act (Stone, 2015; Sinha and Kesselheim, 2016). Established with the intention to streamline antibiotic development, LPAD allows faster access to antibiotics for patients with serious or life-threatening bacterial infections in which no appropriate treatment options exist. The drug’s safety and effectiveness can be studied in significantly smaller, more rapid, and less expensive clinical trials using this mechanism, which is similar to the orphan drug approval process (Simoens et al., 2012; Kwok and Koenigbauer, 2015). Data considered acceptable for drug approval using this pathway can include a combination of non-clinical, *in vitro* susceptibility, pharmacokinetic-pharmacodynamic, and phase II data (Rex et al., 2013). An antibiotic approved by this pathway must have “Limited Population” in the labeling of the drug.

The GAIN act and LPAD pathway are important milestones for revitalizing the antibiotic pipeline. However, some remain skeptical about the benefits of these expedited programs and have concern that speed is being favored over safety. Critics argue that expedited approval has been granted for drugs that did not meet qualifying criteria (i.e. life-threatening/serious, urgent unmet medical need, breakthrough) and have also pointed out a lack of oversight (or enforcement) of post-marketing surveillance studies for approvals based on surrogate markers (Herper; Frank et al., 2014; Kesselheim and Darrow, 2015; Kesselheim et al., 2015; Kim and Prasad, 2016; Chary and Pandian, 2017; Mostaghim et al., 2017). Others have questioned how safety and efficacy can adequately be assessed from such limited data (Light and Lexchin, 2015).

Streamlining drug approval processes is an important strategy to help bring new antibiotics to market in a shorter timeline, but this is only one of several measures needed to combat antibiotic resistance. Several national and international initiatives aimed at incentivizing antibiotic R&D are currently underway, including tax credits, market exclusivity extension, public-private partnerships, and reimbursements (Brogan and Mossialos, 2016; Sciarretta et al., 2016; Luepke and Mohr, 2017; Luepke et al., 2017). Still, despite an expanding antibiotic pipeline, experts remain concerned that these measures will simply not be enough and that we will be outmatched by worsening antibiotic resistance rates (Antibacterial agents in clinical development: an analysis of the antibacterial clinical development pipeline, including tuberculosis 2017; Breaking throught the wall: A call for concerted action on antibiotics research and development, 2017; Simpkin et al., 2017).

## Drug Repurposing

### Overview

As of September 2017, 48 new antibiotics were in Phase I to Phase III development (Antibiotics currently in global clinical development, 2018). While this news was initially encouraging, further investigation revealed a more sobering outlook. First, only approximately 20-30% will translate to a marketable product, given the success rates of an antibiotic moving through development (Payne et al., 2007; Thomas et al., 2017). Second, most of these antibiotics do not have a novel mechanism of action but are instead modifications of existing antibiotic classes (Antibiotics currently in global clinical development, 2018). Third, only 38% of the antibiotics in development are expected to be active against ESKAPE pathogens (*Enterococcus faecium*, *Staphylococcus aureus*, *Klebsiella pneumoniae*, *Acinetobacter baumanii*, *Pseudomonas aeruginosa*, and *Enterobacter*), which have been regarded as high priority for more than a decade (Boucher et al., 2009; Thomas et al., 2017). These factors, combined with the efficacy and safety uncertainties of expedited/LPAD pathway approvals leave little optimism for the future of antibiotics.

Perhaps an alternative solution to the scientific, regulatory, and safety barriers may be to seek new therapeutic uses from existing drugs, also known as drug repurposing or drug repositioning (Beachy et al., 2014). This is becoming an increasingly attractive translational strategy to expedite therapies into the clinic, circumventing much of the early phases of drug development. Drug repurposing is, in itself, an expedited process.

Drug repurposing provides a way for pharmaceutical companies to reduce costs, increase efficiency, and minimize investment and safety risks (Tobinick, 2009; Strittmatter, 2014; Pryor and Cabreiro, 2015). Developing a repurposed drug, as opposed to a newly discovered compound, has the potential to save more than $1 billion and reduce the time to FDA approval by 50% (Scannell et al., 2012; Beachy et al., 2014). Using this strategy can also bring a failed drug back to life, often in the form of a different indication, thereby adding value back to a lost investment.

There is no ‘standardized’ definition for drug repurposing. However, the term does have many other names, which has caused some confusion: “repositioning”, “reprofiling”, “redirecting”, “rediscovery”, and “redeployment”, to name a few (Langedijk et al., 2015). These terms are generally considered interchangeable, with the exception of one – “drug rescue.” This is specifically applied to drugs that failed their intended indication and have successfully been developed for an unrelated indication (Cavalla and Singal, 2012; Medina-Franco et al., 2013). There is also some disagreement as to whether the terms “drug repurposing” and “drug repositioning” are synonymous. The source of some of this confusion is that the wording does not indicate whether a drug failed, was withdrawn, or was abandoned (i.e., early versus late development stages). Even the wording “existing drugs” is not very clear, since this could be interpreted as drug candidates in development or FDA approved drugs. Clearly, the terminology is a work in progress. Dr. Hermann Mucke, PhD, editor of Drug Repurposing, Rescue, and Repositioning, suggested that the term “drug repurposing” be used as a ‘catch-all’ phrase to describe the general concept of “developing an active pharmaceutical ingredient, at any stage of the life cycle and regardless of the success or misfortune it has encountered so far; to serve a therapeutic purpose that is significantly different from the originally intended one.” (Mucke, 2017) For the sake of simplicity, we will follow this suggestion and use the wording “drug repurposing” as an all-encompassing term.

Drug repurposing is not a new concept. In fact, it accounts for approximately 30% of all FDA approved drugs in recent years (Jin and Wong, 2014). Repurposing is a rapidly emerging field, galvanizing interest from both industry sponsors and government agencies. In 2012, the National Center for Advancing Translational Sciences (NCATS) launched a drug repurposing program, entitled “Discovering New Therapeutic Uses for Existing Molecules.” (Allison, 2012) Recognizing the value of drug repurposing, as well as the value of public-private collaborations, NCATS established a three-way partnership between academia, industry, and government with the goal to identify new therapeutic uses of propriety assets (drugs/biologics) across a range of human diseases in areas of unmet clinical need. Drug repurposing partnerships are established through the use of template agreements. AstraZeneca, Janssen Research, Pfizer, and Sanofi are a few of the companies making their assets available to researchers. Essentially, NCATS acts as the ‘matchmaker’ between the ideas from academia and experimental assets from pharmaceutical companies.

Successful drug repurposing may lead to three possible outcomes: (1) new indications for shelved candidates, (2) line extension for existing drugs, and (3) new targets and new indications for existing drugs (Tobinick, 2009; Beachy et al., 2014). Shelved drugs may have failed due to efficacy or safety reasons or were discontinued by the sponsor for strategic reasons. ‘Existing drugs’ refers to those that are FDA approved and currently available. Drugs for which there is pre-existing knowledge of safety and toxicity data (i.e. cleared Phase I trials) are generally the most ideal candidates for repurposing, as this significantly de-risks the clinical development phases. Furthermore, sponsors can leverage pre-existing safety and/or efficacy data to streamline the regulatory approval process of their drug through another expedited pathway, known as the 505(b)(2) pathway. Established in 1984, this pathway is used when changes have been made to previously approved drugs. Ceftazidime-avibactam, which is a combination of an approved cephalosporin (ceftazidime) and a novel β-lactamase inhibitor (avibactam) was approved in 2015 using the 505(b)(2) pathway (Hwang and Kesselheim, 2016). Ceftazidime-avibactam was also a QIDP designated drug, therefore receiving priority review, fast-track designation, and an additional five years of exclusivity.

Repurposing has led to a number of successful drug launches (**Table 4**) (Padhy and Gupta, 2011; Sardana et al., 2011; Barratt and Frail, 2012; Carlson-Banning et al., 2013; DiMasi, 2013; Beachy et al., 2014; Heslop et al., 2015; Rumore, 2016). Many of these success stories were the result of serendipitous re-discoveries, having once been abandoned or shelved after failing during early development for the intended indication. Zidovudine, for example, was originally developed as an anticancer agent, but development came to a halt after testing in animal models was unsuccessful. Years later, zidovudine was found to have potent *in vitro* activity against HIV, and after a rigorous clinical trial, was fast-tracked to FDA approval in 1987 (Yarchoan and Broder, 1987). This marked the beginning of the antiretroviral era, paving the way for discovery of additional life-saving antiretroviral drugs (Broder, 2010). Probably the most notable example of reviving a discontinued drug is the case of thalidomide. Originally marketed in 1957 to pregnant women for treatment of morning sickness, thalidomide was found to have devastating teratogenic effects, causing more than 10,000 birth defects (McBride, 1976; Vargesson, 2015), and was withdrawn in 1961. Thalidomide resurfaced in the late 1990s due to growing interest in the drug for treatment of erythema nodosum leprosum, an indication for which it received FDA approval in 1998 (pregnant women were excluded from study population). This launched an aggressive effort by the sponsor (Celgene) to pursue additional indications and to develop analogues that lacked the teratogenic side effects. In 2003, thalidomide was also approved for treatment of multiple myeloma and quickly became Celgene’s blockbuster drug (Novac, 2013). The thalidomide case, a redeployment of a once considered disastrous drug, demonstrates that repurposing possibilities can arise even from the unlikeliest of sources. This fuels hope that similar repurposing successes will be possible with antimicrobials.

**Table 4:** Examples of drugs successfully repurposed or repositioned.

Drug	Original Indication	New Indication
Amantadine	Influenza	Parkinson’s disease
Amphotericin B	Fungal infections	Leishmaniosis
Aspirin	Inflammation, pain	Antiplatelet
Duloxetine	Depression	Fibromyalgia
Finasteride	Prostate hyperplasia	Hair loss
Gabapentin	Epilepsy	Neuropathic pain
Minoxidil	Hypertension	Hair loss
Thalidomide	Morning sickness	Leprosy, multiple myeloma
Sildenafil	Angina	Pulmonary hypertension, erectile dysfunction
Zidovudine	Cancer	HIV/AIDS

### Non-Antibiotics

No drugs to date have been repurposed as antibiotics. However, a number of existing drugs have demonstrated *in vitro* activity against bacterial pathogens and are therefore known as “non-antibiotics.” They exist among a range of drug classes (Mazumdar et al., 2010; Carlson-Banning et al., 2013; Enserink, 2014; Perlmutter et al., 2014; Thangamani et al., 2015; Schneider et al., 2017; Stewart, 2015; Ruiz et al., 2017), and their microbiological activity is highly variable depending on the specific drug. In many cases, non-antibiotic concentrations used during *in vitro* testing exceed human plasma levels (Sun, 2015). In those instances, it is unlikely a non-antibiotic would be pursued (alone) as a therapeutic agent, given the lack of potent *in vitro* activity. That being said, plasma concentration is not the only metric for drug exposure. Drugs with a large volume of distribution have more drug distributed into the tissues than in plasma and could be useful for soft-tissue infections rather than bloodstream infections.

### Antibiotic Adjuvants

Whether or not a non-antibiotic demonstrates antibacterial activity, directly, as monotherapy, is only one part of the story. Antibacterial properties of a drug can also be apparent when in combination with an antibiotic. When presence of a non-antibiotic drug enhances the *in vitro* activity of an antibiotic, that drug is referred to as an “antibiotic adjuvant” or “antibiotic potentiator.” (Ejim et al., 2011) Some investigators refer to them as “antibiotic helper compounds,” “resistance modulators,” or “resistance breakers.” (Gibbons and Udo, 2000; Mazumdar et al., 2010; Bernal et al., 2013; Brown, 2015; Stenger et al., 2015; de Araujo et al., 2016) Adjuvants not only potentiate antibiotic activity, but they can also minimize or even prevent antibiotic resistance (Kalan and Wright, 2011; Gill et al., 2015).

Use of combination regimens for the prevention or reversal of antibiotic resistance is already a common strategy, but is typically performed with the combination of two antibiotics, rather than an antibiotic plus antibiotic adjuvant. The goal with co-administration of two antibiotics is to achieve synergistic activity, or when the sum of *in vitro* activity of two drugs is greater than with either agent alone (Pillai et al., 2005). The ideal scenario is to administer reduced doses of both antibiotics to decrease the risk of toxicity. However, given the increasing presence of multi-drug resistant pathogens (e.g., *Pseudomonas aeruginosa*), this is not a realistic treatment approach. Though it is possible that one of the combined antibiotics could be administered at a reduced dose, this applies only to certain types of infections and/or specific bacterial pathogens, such as the use of ampicillin with low-dose gentamicin for enterococcal endocarditis (Le and Bayer, 2003).

An antibiotic plus antibiotic adjuvant combination differs from antibiotic-antibiotic combination in that the adjuvant itself may have little to no *in vitro* activity against bacteria, especially at clinically relevant doses (Kalan and Wright, 2011; Gill et al., 2015). The primary action of the adjuvant is to enhance antibiotic activity. Gill et al. has characterized adjuvant mechanisms by dividing them into anti-resistance and anti-virulence (Gill et al., 2015). Quorum sensing inhibitors have demonstrated some success *in vitro* and in murine models for treatment and prevention of biofilm formation (Chow et al., 2014; Sully et al., 2014). Meanwhile several anti-toxin antibodies are undergoing clinical trials. Efflux pumps have become a source of considerable research, particularly the NorA efflux pump that confers a multidrug resistant phenotype of *S. aureus* (Gibbons et al., 2003; Couto et al., 2008; Felicetti et al., 2017; Zimmermann et al., 2017).

## Conclusion

Unfortunately, drug development is notoriously riddled with barriers and has a high failure rate (DiMasi, 2014; DiMasi et al., 2016). One of the major benefits of drug repurposing is that it can significantly reduce this lengthy timeline, thereby making treatments more readily available for clinical unmet needs (Ashburn and Thor, 2004; Barratt and Frail, 2012; Austin and Gadhia, 2017). Drug repurposing is simply a more expedited form of traditional drug development.

The conventional understanding of translational science is that it is a “bench-to-bedside” forward movement that begins in a laboratory and ends with the treatment of patients (Fernandez-Moure, 2016; Fort et al., 2017). Not only is drug repurposing a translational process, but it is also the embodiment of a concept known as “reverse translation.” This is defined loosely as the use of scientific research from an earlier phase to answer questions arising from clinical data (Shakhnovich, 2018). Reverse translation typically begins with patients, in which a question is identified during patient care experiences or clinical trials, and works backwards to test the new hypothesis. Reverse translation is therefore often referred to as a “bedside-to-bench” approach, as it occasionally requires revisiting basic sciences to answer a clinically relevant question (Becker and Funk, 2018). The same principle applies with drug repurposing, in which existing drugs are reevaluated for new indications. Drugs that tend to have the highest probability of success and shortest turnaround time to a new indication are those that are reevaluated within the clinical development phases (i.e., T1 - T3) that have at least cleared phase I clinical trials (i.e., safety and tolerability). Even drugs that failed to meet clinical efficacy endpoints for their desired indications can be excellent candidates for repurposing.

Discovery of an unanticipated drug effect (e.g., a side effect or therapeutic effect unrelated to the primary indication) occurs most commonly during patient care. Investigation of this unexpected effect is a frequent driver for working backwards to reevaluate an old drug. Though it is more ideal to stay within the T1-T3 phases during reevaluation, many of these clinically relevant questions require investigations at the basic science level (Becker and Funk, 2018; McWilliam et al., 2018).

The traditional process of drug discovery and development of a novel compound has a notoriously low probability of success. Drug repurposing, a reverse translational process, is a strategy that can dramatically increase this probability. Using pre-existing scientific knowledge, usually in the form of human clinical data, drug repurposing has the potential to turn even a failed drug into a success.

## Author Contributions

Study concept and design: NB and CF. Interpretation of data: all authors. Drafting of the manuscript: NB. Critical revision of the manuscript for important intellectual content: all authors. Study supervision: NB and CF.

## Funding

CF was supported, in part, by an NIH Clinical and Translational Science Award (National Center for Advancing Translational Sciences, UL1 TR002645) while the study was being conducted.

## Disclaimer

The views expressed in this article are those of the authors and do not necessarily represent the views of the Department of Veterans Affairs, the National Institutes of Health, or the authors’ affiliated institutions.

## Conflict of Interest

CF’s institutions have received grant money, for CF to perform research, from AstraZeneca Pharmaceuticals, in the last 3 years.

The remaining authors declare that the research was conducted in the absence of any commercial or financial relationships that could be construed as a potential conflict of interest.
